# Integrated Multiclass Driver ctDNA Profiling Enables MPNST Detection and Monitoring in NF1 Patients

**DOI:** 10.21203/rs.3.rs-7330245/v1

**Published:** 2025-09-30

**Authors:** Paul A. Jones, Aaron U. Bektas, Jeffrey J. Szymanski, R. Taylor Sundby, John S.A. Chrisinger, Peter K. Harris, Mark I. Zoberi, Kara Weekley, Sanita Burgic, Stacey Chamberlain, Faridi Qaium, Jack F. Shern, Aadel A. Chaudhuri, Angela C. Hirbe

**Affiliations:** Washington University School of Medicine; Washington University School of Medicine; Mayo Clinic; Nemours Children’s Health, Lisa Dean Moseley Foundation Institute for Cancer and Blood Disorders; Washington University School of Medicine; Washington University School of Medicine; Washington University School of Medicine; Washington University School of Medicine; Washington University School of Medicine; Washington University School of Medicine; Mayo Clinic; National Cancer Institute, National Institutes of Health; Mayo Clinic; Washington University School of Medicine

## Abstract

We developed and cross-validated an integrated circulating tumor DNA assay incorporating SNVs, indels, CNAs, and SVs to distinguish neurofibromatosis type 1 patients with malignant peripheral nerve sheath tumors from those with benign plexiform neurofibromas or tumor-free controls. Among 82 participants, the assay achieved an AUC of 0.904, compared with 0.735 for genome-wide CNAs. We also detected recurrent disease-specific driver alterations, relapse up to three months before diagnosis, and early clearance consistent with durable remission.

Malignant peripheral nerve sheath tumors (MPNST) are aggressive sarcomas that frequently arise in the context of neurofibromatosis type 1 (NF1), the most common inherited cancer-predisposition syndrome. These tumors are associated with substantial morbidity and an abysmal 5-year overall survival of 16–32% (1), attributable to MPNSTs’ propensity for rapid progression, early metastasis, and frequent post-treatment recurrence. Timely and reliable detection of malignant transformation remains difficult, especially in the context of plexiform neurofibroma. Plexiform neurofibromas (PN) are benign nerve-sheath tumors affecting up to half of individuals with NF1 (2), occurring anywhere in the body and posing significant monitoring challenges. In NF1, MPNST typically evolved within benign PN. These lesions can be difficult to distinguish from malignant transformation based on early clinical or radiologic features. Magnetic resonance imaging (MRI), the standard modality for NF1 surveillance, may suggest malignancy based on size change, enhancement, or internal complexity, but has limited sensitivity for malignant transformation (3). Positron emission tomography (PET)-computed tomography (CT), while more sensitive than standard MRI, lacks specificity and incurs radiation (4). PET is therefore not recommended for screening in patients with NF1 who are already at an increased risk of malignancy. Tissue biopsy, though more conclusive, may fail to capture focal transformation due to spatial heterogeneity or sampling limitations, particularly in deepseated, multifocal, or surgically inaccessible tumors, and carries some risk of nerve injury (5, 6). This diagnostic uncertainty creates barriers to early intervention, delaying treatment decisions and limiting opportunities for curative intent.

Limitations of our current standard-of-care diagnostics (symptomatic PET with confirmatory biopsy) have accelerated interest in noninvasive tools capable of distinguishing MPNST from PN. This has prompted the development of liquid biopsy approaches, particularly those leveraging circulating tumor DNA (ctDNA), which captures fragments of tumorderived DNA in the bloodstream to provide a realtime molecular snapshot and enable detection of genomic alterations without invasive biopsy (7). Such testing is especially valuable in NF1, where patients often remain in a prolonged premalignant state requiring lifelong surveillance (8). All prior studies have relied on untargeted, genomewide strategies, such as assessing copynumber alterations with lowpass wholegenome sequencing (9,10) exploring fragmentomic features of cellfree DNA (cfDNA)(11), or profiling methylation signatures (12) Here, we present the first ctDNA assay for MPNST specifically designed to detect driverassociated alterations at basepair resolution using CAncer Personalized Profiling by deep Sequencing (CAPPSeq) (13). This targeted, highdefinition approach yields detailed base and mutationlevel information, enabling highly sensitive detection of minimal residual disease (MRD) through identification of specific diseaseassociated mutations and precise monitoring of treatment response.

We enrolled 82 participants: 26 with MPNST (10 localized, 16 metastatic), 44 with plexiform neurofibromas (PN), and 12 healthy controls (Supplementary Table 1). PN and control participants each provided a single baseline plasma sample. In the MPNST group, 44 cfDNA samples were collected across multiple timepoints: at diagnosis (*n* = 13), during routine NF1 surveillance or treatment (*n* = 18), and at clinical recurrence (*n*=13). Tumor tissue was available for 19 MPNST tumors, with some cases contributing samples from multiple tumors. cfDNA was isolated and analyzed by CAPP-Seq using a custom hybrid-capture panel spanning approximately 304 kb enriched for specific MPNST-associated alterations (14). The panel included fullgene coverage for key MPNSTassociated tumor suppressors (*CDKN2A/B*, *TP53*, *SUZ12*, and *EED*) (Supplementary Table 2). This panel also included exonic regions of candidate oncogenes on chromosome 8q (15), *NF1* (the key tumor suppressor frequently lost in NF1, secondarily in and PN and MPNST formation), additional genes implicated in MPNST transformation, and loci commonly mutated in clonal hematopoiesis of indeterminate potential (CHIP) (Supplementary Fig. 2). The mean deduplicated ontarget sequencing depth was 1,032× for plasma, 674× for germline, and 300× for tumor (Supplementary Table 3).

All sequencing data were processed using the Pipeline for the Analysis of ctDNA (PACT) (16), a unified workflow for SNV and indel calling, gene-level CNA detection via CNVkit, and structural-variant detection with SURVIVOR. Variants were filtered using allele-fraction thresholds, matched-germline subtraction, a 12-sample healthy-plasma panel, and backgrounderror control models (see methods). SNVs and SVs were encoded as ordinal severity scores based on predicted functional impact (see methods), and CNAs were standardized as gene-level z-scores. For comparison, tumor-fraction estimates were also calculated from off-target reads using ichorCNA (17, 18). When available, tumor DNA and RNA were used for limited orthogonal validation (n = 19). All somatic features were integrated into a multiclass logistic-regression model trained with leave-one-out cross-validation (LOOCV) and compared with ichorCNA-derived tumor fraction.

Analysis of copy-number alterations (CNAs) revealed minimal detectable signal in cfDNA from healthy individuals and PN patients, consistent with the relative genomic stability expected in non-malignant samples (Fig. 1a). In contrast, cfDNA from MPNST patients displayed diverse CNA profiles but with recurrent alterations affecting established tumor-associated regions, including frequent loss of established tumor suppressors (*CDKN2A/B*, *TP53*, and *SUZ12*), gain across chromosome 8q (e.g., *MYC*, *RAD21*, and *TCEA1*), and notable losses at *MTAP* and *NF1*. These plasma CNA patterns broadly reflected those observed in matched tumor samples, reinforcing the relevance of ctDNA as a surrogate for tissue-derived alterations.

Structural variants (SVs) were similarly sparse in healthy and PN cfDNA, with rare calls likely attributable to technical artifacts or background noise (Fig. 1b) due to their calls in intronic regions. In contrast, MPNST cfDNA revealed numerous SVs, including deletions, inversions, and duplications, whose global patterns in plasma broadly recapitulated those observed in tumor DNA, further supporting the malignant specificity of these signals.

Single-nucleotide variants (SNVs) and short indels were largely absent in healthy plasma samples and infrequently observed in PN cfDNA (Fig. 1d). MPNST samples, however, showed a greater burden of both oncogenic and non-oncogenic events. These mutations frequently involved known tumor suppressors and recapitulated alterations observed in matched tumors, underscoring malignant genomic changes. Together, these results support the specificity of ctDNA alterations to malignant transformation and the concordance of cfDNA profiles with tumor biology in NF1-associated MPNST.

To quantify diagnostic performance, we trained a logistic regression model integrating features from SNVs, CNAs, and SVs. The resulting multiclass ctDNA classifier discriminated malignant from non-malignant plasma samples with a leave-one-out cross-validated AUC of 0.904 (95% CI, 0.833–0.976). At the optimal Youden index threshold, the classifier achieved 83.3% sensitivity and 94.2% specificity. Classifier-derived probabilities sharply separated MPNST from PN and healthy cfDNA (*p* = 9.8 × 10^−12^; Fig. 2b). In comparison, ichorCNA-derived tumor fraction showed inferior performance, with an AUC of 0.735 (95% CI, 0.611–0.859), 53.3% sensitivity, and 96.2% specificity, and separation between groups was correspondingly weaker (*p* = 4.4 × 10^−4^; Fig. 2c).

To explore the clinical utility of ctDNA for postoperative surveillance, we performed longitudinal monitoring in MPNST patients using serial plasma sampling to track molecular and tumor dynamics. As an illustrative example, we followed a patient who initially presented with a localized MPNST of the left shoulder and underwent definitive resection with curative intent, assessing ctDNA changes alongside the clinical course. He had no evidence of disease (NED) at the three-month postoperative follow-up. Approximately six months after surgery, acute symptoms from a collapsed lung led to the discovery of widespread metastatic disease. Retrospective plasma analysis revealed a deletion in *TP53* present at resection, undetectable immediately postoperatively, then re-emerging in plasma at the three-month follow-up and again in the recurrence biopsy (Fig. 2d). The associated CNA profile showed a marked transition between postoperative with chr8 gene-gain apparent in the follow up ctDNA, mirroring tumor evolution. RNA sequencing and immunohistochemistry corroborated the presence of this *P53* SV in the recurrence specimen. Molecular relapses were detectable in plasma approximately 80 days before clinical diagnosis, supporting ctDNA surveillance as a complement to imaging and a potential trigger for earlier evaluation.

As a second example, we followed a patient who presented with a large, invasive MPNST of the left chest wall and underwent complete surgical resection with curative intent. This patient remained disease-free for at least 12 months following treatment. ctDNA profiling identified three tumor-informed variants in pretreatment plasma: *TERT*, *CCDC56*, and *NCOA2*. All three became undetectable after surgery and remained absent in subsequent plasma samples, indicating complete molecular clearance and durable remission (Fig. 2e).

Overall, our results demonstrate that ctDNA profiling provides a precise, disease-relevant approach for detecting malignant transformation in NF1. By integrating single-nucleotide variants, insertions/deletions, gene-level copy-number alterations, and structural variants into a unified logistic-regression model, we achieved high discriminatory performance with an AUC of 0.904. This classifier significantly outperformed ichorCNA-derived tumor fraction using off-target reads and more clearly separated MPNST from benign and healthy samples. Beyond initial detection, ctDNA enabled identification of molecular relapse before radiographic recurrence and confirmed variant clearance after curative resection in longitudinal cases (Fig. 2d–e). These applications underscore the clinical relevance of ctDNA for tracking minimal residual disease and highlight its ability to noninvasively capture tumor dynamics over time. Unlike personalized tumor-informed assays that require patient-specific sequencing, our panel-based approach offers a scalable, off-the-shelf method for high-depth ctDNA profiling in NF1-associated MPNST. The targeted panel captures alterations relevant to MPNST, and the unified modeling framework integrates them into a single predictive score, allowing accurate classification without tailoring to individual cases. In clinical settings where imaging may fail to identify malignant progression and biopsy can miss focal transformation, ctDNA provides a practical adjunct for diagnosis, surveillance, and post-treatment monitoring. With broader validation, this multiclass strategy could enable earlier intervention and more individualized patient management.

## Methods

### Pre-analytical Methods (Clinical and Laboratory Processing)

#### Study Design and Patient Enrollment

Biospecimens were prospectively collected between 2016 and 2024 at Washington University in St. Louis (WUSTL) under protocols approved by the Institutional Review Board (IRB 201903142, 201203042). Eligible participants included individuals with a confirmed clinical or radiographic diagnosis of plexiform neurofibromas (PN) and those with biopsy-proven malignant peripheral nerve sheath tumors (MPNST). NF1 diagnoses were established using NIH consensus criteria. Pathological confirmation of MPNST diagnosis was conducted by board-certified sarcoma pathologists. A total of 44 patients with PN and 26 patients with MPNST were enrolled. From the MPNST cohort, 44 serial plasma samples were collected across multiple timepoints to evaluate tumor dynamics. An additional group of 12 healthy participants (with an additional 12 for PACT artifact background polishing) provided blood samples for background modeling. All participants provided written informed consent prior to enrollment.

#### Sample Collection and Processing

Peripheral venous blood (10–30 mL) was collected in K2EDTA tubes (BD Biosciences) and stored at 4°C until processing within 4 hours of phlebotomy. Blood samples underwent initial centrifugation at 1,200 × g for 10 minutes at ambient temperature to separate plasma from cellular components. The plasma supernatant was carefully transferred into DNA low-bind microcentrifuge tubes and subjected to a secondary centrifugation at 1,800 × g for 5 minutes to further deplete cellular debris. Clarified plasma was aliquoted into 1.5 mL microcentrifuge tubes and stored at −80°C until DNA extraction. The residual cellular fraction (plasma-depleted whole blood, PDWB) was also aliquoted and stored at −80°C for germline DNA isolation. Plasma samples exhibiting signs of hemolysis or visible degradation were excluded from further analysis. Tumor tissue was obtained at the time of clinical biopsy or surgery, following institutional pathology workflows. FFPE blocks or snap-frozen specimens were retained for molecular studies if excess diagnostic tissue was available. MPNST and PN samples were sequenced within the same Illumina flow cell lanes to mitigate batch effects.

#### cfDNA and Genomic DNA Extraction

Circulating cell-free DNA (cfDNA) was isolated from 2–8 mL of thawed plasma using the Qiagen Circulating Nucleic Acid Kit in conjunction with the QIAvac vacuum manifold system, following manufacturer protocols. Germline DNA was extracted from PDWB using the Qiagen DNeasy Blood and Tissue Kit. Tumor DNA was isolated using either the AllPrep DNA/RNA FFPE Kit for FFPE sections or the DNeasy kit for frozen tissue specimens. Extracted DNA was quantified using the Qubit dsDNA High Sensitivity Assay (Thermo Fisher Scientific), and fragment size distributions were assessed using the Agilent 2100 Bioanalyzer. To optimize downstream library preparation and hybridization, tumor and germline DNA were fragmented to a modal size of approximately 200 base pairs using a Covaris LE220 focused ultrasonicator. cfDNA underwent no additional shearing. Plasma-derived samples primarily composed of high-molecular weight DNA were excluded from processing. Samples with insufficient cfDNA yield for library preparation were excluded from downstream analysis.

#### Targeted Panel Design and In Silico Validation

A custom hybrid capture panel was developed using the Roche HyperDesign platform to target refined genomic regions totaling ~304 kb. Regions were consolidated from an initial 3,615 candidates to reduce redundancy and improve coverage efficiency. Genes were organized into three tiers. Tier 1 included genes with high recurrence, whole-gene coverage, and relevance to early malignant transformation, particularly *CDKN2A*, *CDKN2B*, *TP53*, *SUZ12*, and *EED*. These regions were selected to support detection of inactivating SNVs, splice-altering variants, and structural variants such as inversions or deletions. Tier 2 included candidate oncogenes located on chromosome 8q and associated with tumorigenesis upon amplification. Tier 3 comprised a mixture of lower-prevalence genes, *NF1*, and genes associated with clonal hematopoiesis of indeterminate potential. Probe selection aimed to maximize specificity and minimize off-target binding. In silico benchmarking was performed using publicly available cancer datasets and the Genomics of MPNST (GeM) cohort.

#### Library Preparation and Sequencing

Library construction was performed using the KAPA HyperPrep Kit (Kapa Biosystems, catalog no. KK8502). cfDNA libraries were prepared from 32 ng of input DNA whenever possible; for samples yielding <32 ng, a minimum of 10 ng of DNA was used for preparation. Libraries underwent 12 cycles of pre-capture PCR, with a subset undergoing up to 15 cycles due to low DNA mass or low post-PCR yield. Tumor and germline libraries were constructed from a similar 32 ng of input DNA. Fragment size was confirmed with the Agilent Bioanalyzer. A total of 1.5 μg of pooled library DNA (12-plex) was used for hybridization capture with the custom panel. Libraries were pooled with equivalent mass input across samples. Following capture, libraries underwent 17 cycles of post-enrichment PCR amplification. Sequencing was performed on an Illumina HiSeq 4000 system (Illumina Inc., San Diego, CA) with 2 × 150 bp paired-end reads and an 8-base indexing read. Mean unique sequencing depth was approximately on-targeted 1032x, 674x, and 300x for cfDNA, Germline and Tumor respectively.

### Analytical Methods (Computational and Statistical Analysis)

#### Read Alignment and Preprocessing

All SNV, small indel, CNA, and SV calling was performed using the Pipeline for the Analysis of ctDNA (PACT), developed at Washington University School of Medicine (Webster et al., *Bioinformatics* 2023). PACT is a unified workflow designed to reproducibly identify single-nucleotide variants, indels, copy number alterations, and structural variants from ctDNA sequencing data. The pipeline integrates multiple variant calling strategies with support for matched controls and unmatched healthy plasma background models to reduce artifacts and increase specificity, particularly for low-frequency ctDNA events. It is distributed as a Common Workflow Language (CWL) implementation and is freely available at https://github.com/ChrisMaherLab/PACT. For this project, paired-end reads were demultiplexed using sample-specific index barcodes and aligned to the hg19 reference genome using BWA-MEM v0.7.17 with default parameters. Alignments were converted to BAM, sorted, and indexed using SAMtools v1.13–1.22. PCR duplicates were identified and removed or marked using either SAMtools markdup or Picard v2.18 as appropriate for downstream CNA, SV, SNV, and indel analyses. Quality control metrics were collected using SAMtools flagstat, FastQC, and BEDTools coverageBed. Only properly paired reads with a mapping quality ≥30 were retained for downstream analysis.

#### SNV and Indel Detection

SNV and small indel detection was performed using the Pipeline for the Analysis of ctDNA (PACT), which integrates an ensemble of Mutect, Strelka, VarScan, and Pindel under relaxed, sensitivity-optimized parameters. Variant calls were normalized using Vt and annotated with VEP. Standardized read counts were computed using bam-readcount, and additional filters were applied based on read depth, mapping quality, allele fraction (AF), population frequency (gnomAD), and signal observed in background controls. Background artifact suppression was implemented by genotyping a preconfigured panel of 12 healthy plasma samples with GATK HaplotypeCaller and removing variants observed in >10% of controls. Variants detected in germline from unrelated individuals were also systematically excluded when consistent with PCR slippage or mismapping artifacts within the same sequencing lane.

Final variant calls were assigned confidence rankings based on caller agreement, read support, AF, and filter annotations. Consequence categories were defined for visualization and classification. Splice variants were grouped as Donor, Acceptor, Region, or Other. Missense variants were labeled as “Protein Disrupting” (PolyPhen damaging and SIFT deleterious), “VUS, “ or “Likely Not Disrupting. “ Truncating loss-of-function (LoF) variants included frameshift, stop-gained, and start-lost events. Diagnostic group, gene identity, and variant confidence were used to control inclusion for downstream analysis.

Within PACT, stringent quantitative thresholds were applied. For cfDNA samples, variants were retained only if they passed all internal PACT filters and had AF > 0.0005 with alternate allele depth ≥8. Tumor variants were included if AF exceeded 0.05 with alternate allele depth ≥8, and were excluded if flagged for contamination, germline origin, or normal artifact. Noncoding variants were not removed but tracked separately for annotation and visualization.

#### Copy Number Alteration Detection

Copy number alterations were detected using CNVkit v0.9.6 within the PACT workflow. Bin-level log_2_ coverage ratios were calculated between cfDNA samples and controls, then corrected for GC content and repeat bias. Ratios were aggregated to gene-level log_2_ values using a trimmed average to reduce the influence of outlier bins. Regions with low plasma read depth or excessive variability were excluded. Gene-level values were normalized using a background reference of expected copy-neutral regions and off-target bins to correct for global technical variation. Per-gene standard deviations were estimated from healthy controls and used to calculate z-scores per sample. *ATRX* (due to X-chromosome effects) and known artifact-prone loci were excluded. CNA log_2_ values and z-scores for *TP53*, *CDKN2A/B*, *SUZ12*, *EED*, and chromosome 8q (averaged across candidate oncogenes) were retained as input features for the integrated classification model. These processed values were also used in violin plots and individual gene-level bar plots

#### Structural Variant Analysis

Structural variants were identified using an ensemble approach involving Delly, Lumpy, and Manta, each run with relaxed parameters to maximize sensitivity. SV candidates were merged using SURVIVOR (100 bp max breakpoint distance, minimum SV size = 200 bp). SVs were retained if supported by ≥2 callers. For cfDNA samples, additional evidence was required in the form of both split reads and discordant pairs (with minimum depth support). Tumor samples were evaluated using the same multi-caller requirement but without the same depth filter. SVs overlapping targeted panel regions were retained; events in blacklist or low-complexity regions were excluded.

All retained SVs were genotyped using SVTyper across samples, controls, and healthy plasma. Events detected in any control were removed. Surviving calls were annotated with SnpEff and formatted in BEDPE. No whitelist was applied. For integrated analysis, a restricted set of high-confidence SVs was used, including disruptions to *TP53*, *SUZ12*, and *EED* (e.g., deletions or complex intronic events) and SVs involving partial or full duplication of chromosome 8.

#### Tumor Fraction Estimation Using ichorCNA

Tumor fraction was estimated for cfDNA samples using ichorCNA v1.0.0 with low TF settings. Prior to coverage binning, reads overlapping the hybrid capture design plus a 500 bp padding window were removed. All remaining reads from canonical autosomes were retained and subsequently downsampled to approximately one million reads. Coverage was computed in 1 Mb bins, and a panel of normals was generated from the twelve healthy cfDNA samples used for background polishing using ichorcna’s inbuilt createpanelofnormals.R. This tumor fraction estimate was used for ROC comparison against the integrated sequencing classifier.

#### cfDNA-Based Multiclass Classification Model

To distinguish malignant peripheral nerve sheath tumors (MPNST) from plexiform neurofibromas (PNs) and healthy controls, we developed a logistic regression classifier integrating multiclass genomic features derived from plasma circulating tumor DNA (ctDNA). Feature classes included copy number alterations (CNAs), single nucleotide variants (SNVs), and structural variants (SVs), each processed to preserve biological relevance and maximize classification performance.

Gene-level CNAs were extracted from targeted sequencing data for *CDKN2A/B*, *TP53*, *SUZ12*, *EED*, and chromosome 8q. For each gene, two metrics were derived: the log_2_-transformed depth ratio and a normalized score calculated by dividing the log_2_ depth ratio by the standard deviation of that gene’s log_2_ depth ratio across the cohort. All CNA features were scaled prior to model inclusion.

SNVs were assigned functional categories in a defined rank order: loss-of-function, protein-disrupting missense, splice-altering, variants of uncertain significance (VUS), non-disrupting missense, synonymous, and other. For each gene, the highest-severity event per sample was retained and encoded on a 0–6 ordinal scale. In addition, a count-based variable was derived for SNVs to classify each gene as having a single or multiple variant events. Structural variants were similarly processed and categorized by gene as none, single, or multiple events. Events with known germline presence in unrelated patients were manually excluded to avoid confounding from sequencing artifacts.

## Supplementary Material

Supplementary Files

This is a list of supplementary files associated with this preprint. Click to download.

• SupplementalData.xlsx

• Supp1.tif

• Supp2.tif

## Figures and Tables

**Figure 1:**
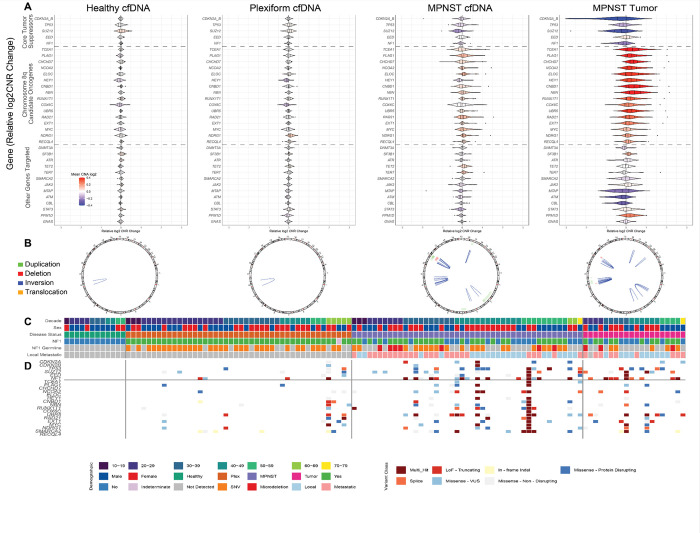
Grand Oncoplot reveals key loss of MPNST-associated tumor suppressors and chromosome 8q candidate oncogenes in plasma, recapitulating alterations found in tumor. A. Copy-number alteration (CNA) landscape. Relative log_2_ copy number ratio (CNR) across healthy cfDNA, PN cfDNA, MPNST cfDNA, and MPNST tumor. Genes are ordered by relevance: (CDKN2A/CDKN2B, SUZ12, TP53, EED, NF1); chromosome 8q candidate oncogenes (e.g., TCEA1, PLAG1, CHCHD7); and less transformation-associated or clonal hematopoiesis–related genes below. MPNST samples show frequent gains and losses at disease-associated loci; PN and healthy cfDNA show minimal alteration. Blue, loss; red, gain. B. Structural-variant (SV) summary. SVs across cfDNA and tumor samples, including deletions, duplications, inversions, or translocations. MPNST cfDNA shows extensive rearrangements recapitulated in matched tumors; PN and healthy cfDNA show few events. One cfDNA sample with likely chromothripsis was excluded. C. Demographic and clinical annotation block. Clinical and demographic metadata per sample, aligned across panels. “Decade” indicates age at blood draw; “Sex” indicates male or female. “Disease status” differentiates healthy cfDNA, PN cfDNA, MPNST cfDNA, and MPNST tumor. NF1 denotes clinical diagnostic status, with “Indeterminate” for individuals with suggestive but insufficient features. NF1 germline mutation and metastasis status (localized vs. metastatic) are also shown. D. Single-nucleotide variant (SNV) and indel oncoplot. Nonsynonymous SNVs and short indels across cfDNA and tumor samples. Gene order matches the CNA panel. Variants are classified by the Precision Analysis for Cancer Tracking (PACT) integrating SIFT/PolyPhen as truncating, splice site, or missense; missense variants are further categorized as protein-disrupting, variant of uncertain significance (VUS), or non-disruptive. Only high-confidence calls are shown; germline, clonal hematopoiesis of indeterminate potential (CHIP)-associated, and low-confidence variants were excluded. “Multi-hit” labels mark genes with multiple events per sample. MPNST samples harbor frequent alterations in core suppressors; PN and healthy samples show few pathogenic mutations. **Abbreviations:** cfDNA, cell-free DNA; CHIP, clonal hematopoiesis of indeterminate potential; CNA, copy number alteration; CNR, copy number ratio; indel, insertion/deletion; MPNST, malignant peripheral nerve sheath tumor; PN, plexiform neurofibroma; SNV, single nucleotide variant; SV, structural variant; VUS, variant of uncertain significance.

**Figure 2:**
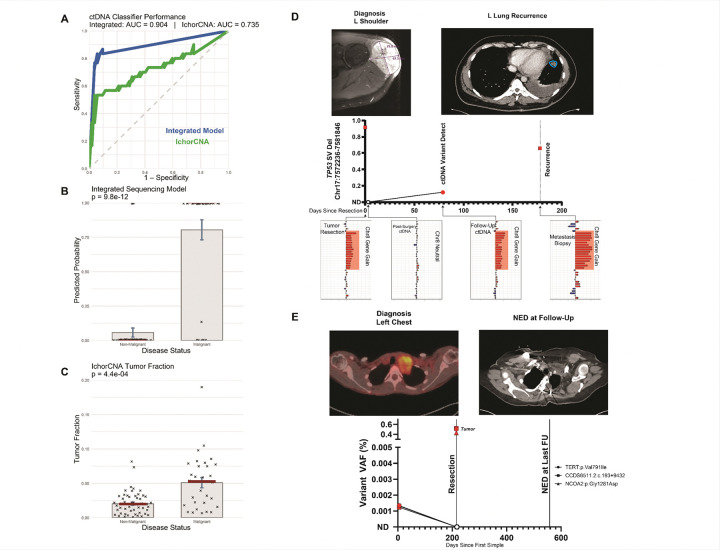
Integrated multiclass circulating tumor DNA (ctDNA) classifier outperforms tumor fraction and enables longitudinal tracking of molecular clearance and recurrence. A. ROC curve. Comparison of the integrated model, integrating single nucleotide variant (SNV), copy number alteration (CNA), and structural variant (SV) features, with ichorCNA-derived tumor fraction. The integrated model achieved a leave-one-out cross-validated (LOOCV) AUC of 0.904, outperforming ichorCNA (AUC = 0.735). All models were trained and evaluated on the same cfDNA cohort. B. Integrated model separation. Predicted malignancy probabilities by disease status. Each point is a cell-free DNA (cfDNA) sample. MPNST and non-malignant groups show clear separation (P = 9.8 × 10^−12^, Wilcoxon rank-sum test). Red lines denote medians; bars show mean ± s.e.m. C. ichorCNA tumor fraction. ichorCNA tumor fraction for the same cfDNA cohort. Group separation remains significant (P = 4.4 × 10^−4^, Welch t test) but is less distinct than the integrated model, highlighting the value of multiclass feature integration. Estimates used off-target reads. D. Longitudinal relapse case. ctDNA tracking at surgical resection, immediate postoperative plasma, three-month follow-up, and recurrence biopsy. A recurrent *TP53* deletion was present at resection, undetectable immediately postoperative, and re-emerged in plasma at the three-month follow-up and in the recurrence biopsy. CNA profiles shifted between postoperative and relapse, mirroring tumor evolution. Molecular relapse was detectable ≈ 80 days before clinical diagnosis. Red symbols indicate variant detected; empty circles indicate variant not detected. E. Longitudinal clearance case. ctDNA profiling in a patient with localized MPNST. Three tumor-informed variants (*TERT*, *CCDC56*, *NCOA2*) were detected in tumor and pretreatment plasma but were absent from postoperative and subsequent plasma samples, indicating complete molecular clearance. The patient remained disease-free for ≥ 12 months. Red symbols indicate variant detected; empty circles indicate variant not detected. **Abbreviations:** AUC, area under the curve; CNA, copy number alteration; cfDNA, cell-free DNA; ctDNA, circulating tumor DNA; ichorCNA, tumor fraction estimation from copy number analysis; LOOCV, leave-one-out cross-validation; MPNST, malignant peripheral nerve sheath tumor; PN, plexiform neurofibroma; ROC, receiver operating characteristic; s.e.m., standard error of the mean; SNV, single nucleotide variant; SV, structural variant.

## Data Availability

Raw and processed sequencing data from this study are available via the Synapse database (https://nf.synapse.org/). A DOI will be provided prior to final publication.

## Code availability

The PACT pipeline used for somatic variant detection is publicly available at https://github.com/ChrisMaherLab/PACT. Tumor fraction estimation was performed using a lightly modified version of ichorCNA available at https://github.com/jeszyman/ichorCNA-patched, based on the original release at https://github.com/broadinstitute/ichorCNA. Custom scripts for feature integration and visualization are available from the corresponding author upon reasonable request.
